# Supplementary material for: Recombination alters the receptor binding and furin cleavage site in novel bat-borne HKU5-CoV-2 coronavirus
Source: Microbiol Spectr. 2025 Aug 29;13(10):e01420-25. doi: 10.1128/spectrum.01420-25 (PMC12502745; doi:10.1128/spectrum.01420-25)
Supplement: Supplemental materials — and methods, Fig. S1, and Tables S1. [file spectrum.01420-25-s0001.docx]

**Supplementary Information**

**Materials and Methods**

Genomic sequences of HKU-CoV-2 (BtHKU5-CoV-2-153, BtHKU5-CoV-2-155, BtHKU5-CoV-2-023, BtHKU5-CoV-2-028, BtHKU5-CoV-2-381, and BtHKU5-CoV-2-441) were obtained from Genbase database (National Genomics Data Center, China, <https://ngdc.cncb.ac.cn/genbase/?lang=en>). Accession numbers: C_AA085189.1, C_AA085190.1, C_AA085191.1, C_AA085192.1, C_AA085193.1, and C_AA085194.1 (1). The nucleotide and SNP number are based on the C_AA085191.1 sequence in this study. Sequence alignment and linkage disequilibrium (LD) analysis using HaploView (https://www.broadinstitute.org/haploview/haploview) are previously described (2, 3). Only mutations shown in the same allele in more than two sequences, but not the singletons, were considered SNPs and analyzed in this study. The *D*’ value was calculated by normalizing the degree of nonrandom association between two alleles (*D*), with its maximum possible value *D*_max_. Haplotype blocks with recombination measurements were determined with the solid spine of LD (3).

The viral sequences were analyzed by Recco program, which scores the cost of obtaining one of the sequences from the others by mutation and recombination (4). Recombinant fragments and breakpoints were measured by parameter α, weighting recombination cost against mutation cost. Only the fragments with significant mutation cost (>5) and *p* value <0.05 were considered as recombinant fragments.

Protein structure of BtHKU5-CoV-2-441 RBD and human ACE2 (PDB: PDB: 9JJ6) are downloaded from RCSB (1). *In silico* computer simulation of T498 and N503 substitution was performed with AlphaFold 3.0.1 and presented by PyMOL (5, 6). Default settings for PyMOL version 3.1.3 were chosen. Besides automatically chosen miniamal steric strain rotamers for substituted amino acids, no other energy minimizations were applied.

**References**

1. Chen J, Zhang W, Li Y,  et al. Bat-infecting merbecovirus HKU5-CoV lineage 2 can use human ACE2 as a cell entry receptor. Cell 2025; 188:1729-1742.e16.
2. Yeh TY, Feehley PJ, Feehley MC, Chen CF, Tsai TY, Cheng HL, Contreras GP. Rapidly evolving recombinant monkeypox virus strains compound the challenges of the 2024 mpox outbreak. Lancet Microbe. 2025; 6:101017.
3. Barrett JC, Fry B, Maller J, Daly MJ. Haploview: analysis and visualization of LD and haplotype maps. Bioinformatics. 2005; 21:263–265.
4. Maydt J, Lengauer T. Recco: recombination analysis using cost optimization Bioinformatics. 2006; 22:1064-1071.
5. Abramson J, Adler J, Dunger J, Evans R, Green R, et al. Accurate structure prediction of biomolecular interactions with AlphaFold 3. Nature 2024; 630:493–500.
6. The PyMOL Molecular Graphics System, Version 1.2r3pre, Schrödinger, LLC.

**Supplemental figure and table legend**

Figure S1. Haplotype block organization of the full-length HKU5-CoV-2 sequences. SNP numbers associated with the numbers above haploblocks is listed in Table S4.

Table S1. Recco analysis of HKU5-CoV-2 sequences*. The recombination breakpoints are verified by LD (recombinant SNP pairs^#^) and haploblock analysis (between or at the end of haploblocks).

Table S2 and S3. The SNP positions (L1 and L2), squared coefficient of correlation (*r*^2^), the logarithm of the odds (LOD), confidence interval (high and low; CIhigh and CIlow), and distance between L1 and L2 (dis) of total (Table S2) and recombinant SNP pairs (Table S3) of HKU5-CoV-2 (Figure 1A and 1B).

Table S4. SNP numbers associated with the numbers above the haploblocks for Figure S1.

Table S5. Summary of recombination breakpoints in HKU5-CoV-2 in Figure 1C. N, numbers of recombinant SNP pairs; N/A, not applicable; S, synonymous mutation; RDRP, RNA-dependent RNA polymerase; MTase, methyltransferase; NTD, N terminal domain; RBD, receptor binding domain; SD, SD-1 and SD2 domain; FCS, furin cleavage site; TM, transmembrane domain, Tail, cytoplasmic tail. The breakpoints located at the haploblock end are marked with X in the Margin.

Figure S1


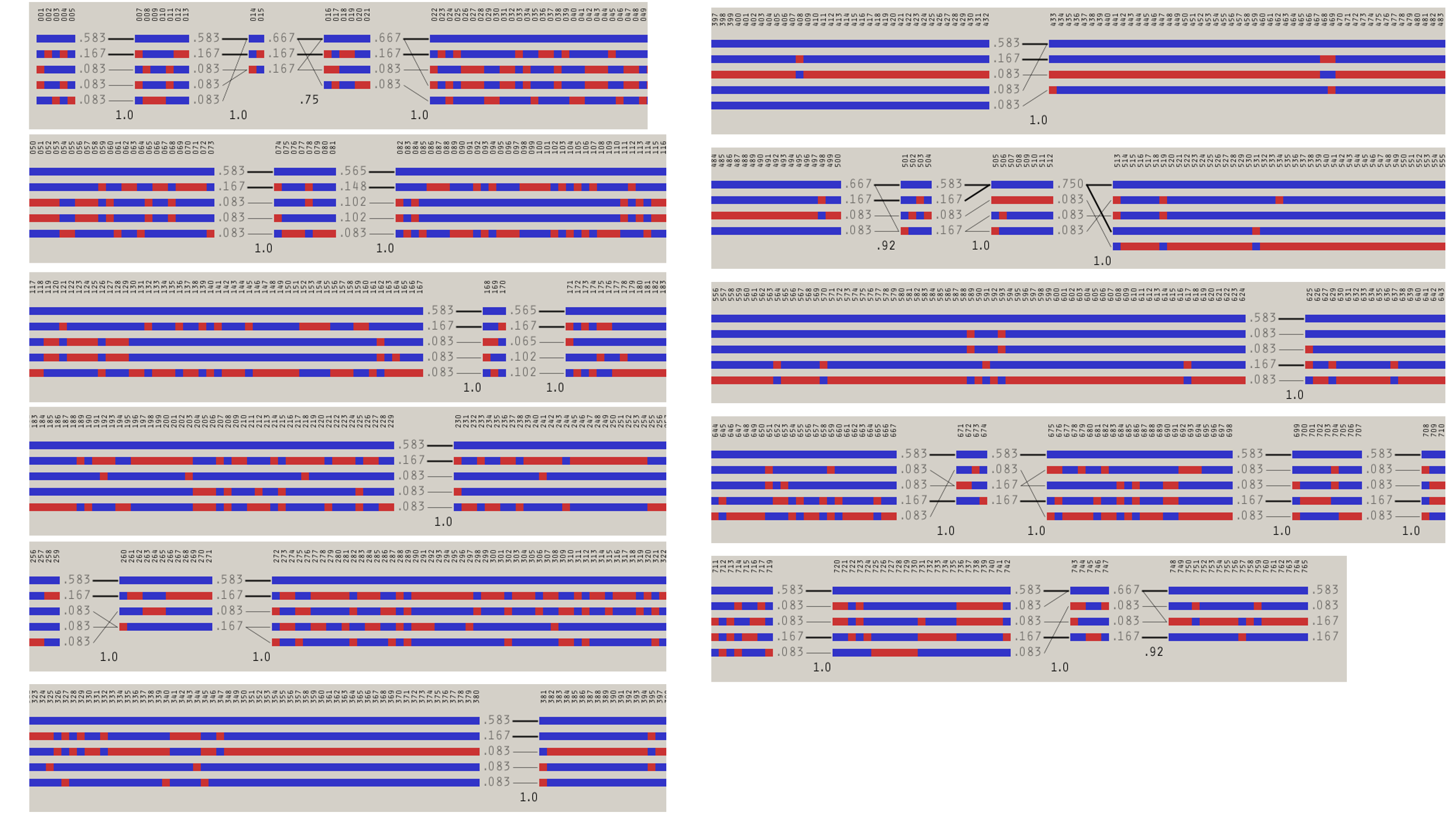


Table S1. Recco analysis of HKU5-CoV-2 sequences*. The recombination breakpoints are verified by LD (recombinant SNP pairs^#^) and haploblock analysis (between or at the end of haploblocks).

| **Recombinant fragment (nucleotide)*** | **Mutation cost*** | **SNP (recombinant SNP pair**  **numbers)^#^** | **Location** | **Haploblock** |
| --- | --- | --- | --- | --- |
| 8893-8997 | 381.2 (*p*=0.04) | 8892 (0) | Polyprotein 1ab, AA 2879-2913, NSP4 transmembrane domain | 10 |
| 18066-18397 | 410.2 (*p*<10^-3^) | 18065 (86) | Polyprotein 1ab, AA 5937-6046, NSP13 Helicase, NSP14 ExoN domain | 12 |
| 19206-19292 | 410.2 (*p*<10^-3^) | 19205 (9) | Polyprotein 1ab, AA 6317-6345, NSP14, N7-MTase active site | 12 (end) |
| 25522-25563 | 394.2 (*p*=0.017) | 25521 (41) | spike protein AA 1286-1299, transmembrane domain | 19, 20 |

Table S5

| **SNP** | **N** | **Protein** | **Residue** | **Mutation** | **Domain** | **Haploblock** | **Margin** |
| --- | --- | --- | --- | --- | --- | --- | --- |
| 127 | 41 | 5' UTR | N/A | N/A | N/A | 1 |  |
| 247 | 36 | 5' UTR | N/A | N/A | N/A | 1 |  |
| 363 | 9 | ORF1ab | L35 | S | NSP1 | Between |  |
| 385 | 36 | ORF1ab | L43 | S | NSP1 | 2 | X |
| 447 | 116 | ORF1ab | L63 | S | NSP1 | 2 |  |
| 495 | 41 | ORF1ab | F79 | S | NSP1 | 2 |  |
| 597 | 41 | ORF1ab | S113 | S | NSP1 | 3 | X |
| 630 | 9 | ORF1ab | Q124 | S | NSP1 | 4 | X |
| 645 | 115 | ORF1ab | G129 | S | NSP1 | 4 |  |
| 777 | 41 | ORF1ab | I173 | S | NSP1 | 5 | X |
| 867 | 87 | ORF1ab | Y203 | S | NSP2 | 5 |  |
| 1090 | 41 | ORF1ab | F278 | Val | NSP2 | 5 |  |
| 1113 | 41 | ORF1ab | P285 | S | NSP2 | 5 |  |
| 1121 | 41 | ORF1ab | C288 | Tyr | NSP2 | 5 |  |
| 1184 | 41 | ORF1ab | G309 | Glu | NSP2 | 5 |  |
| 1218 | 41 | ORF1ab | F320 | S | NSP2 | 5 |  |
| 1353 | 41 | ORF1ab | Y365 | S | NSP2 | 5 |  |
| 1476 | 41 | ORF1ab | T406 | S | NSP2 | 5 |  |
| 1542 | 41 | ORF1ab | V428 | S | NSP2 | 5 |  |
| 1560 | 41 | ORF1ab | C434 | S | NSP2 | 5 |  |
| 1581 | 41 | ORF1ab | F441 | S | NSP2 | 5 |  |
| 1638 | 41 | ORF1ab | C460 | S | NSP2 | 5 |  |
| 1683 | 41 | ORF1ab | L275 | S | NSP2 | 5 |  |
| 1779 | 41 | ORF1ab | E507 | S | NSP2 | 5 |  |
| 1818 | 41 | ORF1ab | I520 | S | NSP2 | 5 |  |
| 1821 | 41 | ORF1ab | R521 | S | NSP2 | 5 |  |
| 1863 | 41 | ORF1ab | S535 | S | NSP2 | 5 |  |
| 2001 | 41 | ORF1ab | L581 | S | NSP2 | 5 |  |
| 2040 | 41 | ORF1ab | Y594 | S | NSP2 | 5 |  |
| 2052 | 41 | ORF1ab | P598 | S | NSP2 | 5 |  |
| 2076 | 41 | ORF1ab | N606 | S | NSP2 | 5 |  |
| 2177 | 41 | ORF1ab | S640 | Phe | NSP2 | 5 |  |
| 2190 | 41 | ORF1ab | S644 | S | NSP2 | 5 |  |
| 2256 | 36 | ORF1ab | F666 | S | NSP2 | 6 | X |
| 2487 | 9 | ORF1ab | Y743 | S | NSP2 | 6 |  |
| 2569 | 41 | ORF1ab | G771 | Ser | NSP2 | 7 | X |
| 2716 | 41 | ORF1ab | L820 | S | NSP2 | 7 |  |
| 4672 | 41 | ORF1ab | A1472 | Ser | NSP3 | 7 |  |
| 5052 | 41 | ORF1ab | A1598 | S | NSP3 | 7 |  |
| 5142 | 41 | ORF1ab | Y1628 | S | NSP3 | 7 |  |
| 5400 | 41 | ORF1ab | L1714 | S | NSP3 | 7 |  |
| 5658 | 41 | ORF1ab | S1800 | S | NSP3 | 7 |  |
| 5761 | 41 | ORF1ab | S1835 | Ala | NSP3 | 7 |  |
| 5911 | 41 | ORF1ab | N1885 | Asp | NSP3 | 7 |  |
| 5922 | 41 | ORF1ab | L1888 | S | NSP3 | 7 |  |
| 5931 | 41 | ORF1ab | F1891 | S | NSP3 | 7 |  |
| 5969 | 41 | ORF1ab | A1904 | Val | NSP3 | 7 |  |
| 6028 | 41 | ORF1ab | V1924 | Ile | NSP3 | 7 |  |
| 6039 | 41 | ORF1ab | D1927 | S | NSP3 | 7 |  |
| 6072 | 41 | ORF1ab | S1938 | S | NSP3 | 7 |  |
| 7491 | 41 | ORF1ab | F2411 | S | NSP3 | 7 |  |
| 7539 | 87 | ORF1ab | Y2427 | S | NSP3 | 7 |  |
| 7870 | 41 | ORF1ab | L2538 | Val | NSP3 | 8 | X |
| 7986 | 116 | ORF1ab | C2576 | S | NSP3 | 8 |  |
| 8400 | 9 | ORF1ab | T2714 | S | NSP3 | 9 | X |
| 8508 | 36 | ORF1ab | T2750 | S | NSP3 | 9 |  |
| 8571 | 87 | ORF1ab | D2771 | S | NSP3 | 9 |  |
| 9128 | 9 | ORF1ab | T2957 | Ile | NSP4 | 9 |  |
| 9903 | 9 | ORF1ab | V3215 | S | NSP4 | 9 |  |
| 10074 | 87 | ORF1ab | Y3272 | S | NSP4 | 9 |  |
| 10233 | 87 | ORF1ab | V3325 | S | Nsp5 | 9 |  |
| 10317 | 87 | ORF1ab | L3353 | S | Nsp5 | 9 |  |
| 10366 | 87 | ORF1ab | L3370 | S | Nsp5 | 9 |  |
| 10689 | 87 | ORF1ab | E3477 | S | Nsp5 | 9 |  |
| 11184 | 87 | ORF1ab | F3642 | S | NSP6 | 9 |  |
| 11565 | 9 | ORF1ab | A3769 | S | NSP6 | 9 |  |
| 12342 | 87 | ORF1ab | L4028 | S | NSP8 | 9 |  |
| 13159 | 36 | ORF1ab | L4301 | S | NSP9 | 10 | X |
| 14111 | 9 | ORF1ab | V4618 | S | RDRP | 10 |  |
| 15353 | 41 | ORF1ab | A5032 | S | RDRP | 11 | X |
| 16088 | 87 | ORF1ab | D5277 | S | RDRP | 12 | X |
| 16112 | 9 | ORF1ab | D5285 | S | RDRP | 12 |  |
| 16125 | 9 | ORF1ab | L5290 | S | RDRP | 12 |  |
| 16223 | 87 | ORF1ab | T5322 | S | RDRP | 12 |  |
| 16238 | 8 | ORF1ab | P5327 | S | RDRP | 12 |  |
| 16274 | 9 | ORF1ab | Y5339 | S | RDRP | 12 |  |
| 16322 | 9 | ORF1ab | Y5355 | S | RDRP | 12 |  |
| 16349 | 87 | ORF1ab | S5364 | S | RDRP | 12 |  |
| 16427 | 9 | ORF1ab | V5390 | S | NSP13 | 12 |  |
| 16433 | 9 | ORF1ab | C5392 | S | NSP13 | 12 |  |
| 16481 | 87 | ORF1ab | F5408 | S | NSP13 | 12 |  |
| 16604 | 9 | ORF1ab | L5449 | S | NSP13 | 12 |  |
| 16637 | 87 | ORF1ab | P5461 | S | NSP13 | 12 |  |
| 16676 | 9 | ORF1ab | F5474 | S | NSP13 | 12 |  |
| 16734 | 9 | ORF1ab | L5493 | S | NSP13 | 12 |  |
| 16772 | 9 | ORF1ab | T5505 | S | NSP13 | 12 |  |
| 17018 | 9 | ORF1ab | I5587 | S | NSP13 | 12 |  |
| 17162 | 87 | ORF1ab | G5635 | S | NSP13 | 12 |  |
| 17507 | 9 | ORF1ab | T5750 | S | NSP13 | 12 |  |
| 17721 | 87 | ORF1ab | L5822 | S | NSP13 | 12 |  |
| 17921 | 87 | ORF1ab | P5888 | S | NSP13 | 12 |  |
| 18065 | 87 | ORF1ab | T5933 | S | NSP13 | 12 |  |
| 18911 | 87 | ORF1ab | V6218 | S | NSP14 | 12 |  |
| 19205 | 9 | ORF1ab | N6316 | S | NSP14 | 12 |  |
| 19322 | 36 | ORF1ab | F6355 | S | NSP14 | 12 |  |
| 20645 | 87 | ORF1ab | V6796 | S | NSP15 | 12 |  |
| 21209 | 9 | ORF1ab | D6984 | S | MTase | 12 |  |
| 21215 | 87 | ORF1ab | C6986 | S | MTase | 12 |  |
| 21879 | 41 | Spike | S72 | S | NTD | 13 | X |
| 22110 | 9 | Spike | P149 | S | NTD | 13 |  |
| 22326 | 87 | Spike | D221 | S | NTD | 14 | X |
| 22597 | 36 | Spike | H312 | Tyr |  | 14 |  |
| 23016 | 41 | Spike | P451 | S | RBD | 15 | X |
| 23043 | 87 | Spike | F460 | S | RBD | 16 | X |
| 23064 | 115 | Spike | S467 | S | RBD | 16 |  |
| 23156 | 41 | Spike | V498 | Thr,Ile | RBD | 17 | X |
| 23193 | 41 | Spike | P510 | S | RBD | 17 |  |
| 23285 | 87 | Spike | V541 | Ala | RBD | 17 |  |
| 23587 | 41 | Spike | V642 | Ile | SD | 17 |  |
| 23625 | 41 | Spike | F654 | S | SD | 17 |  |
| 23833 | 9 | Spike | F724 | Leu | FCS | 18 | X |
| 23847 | 41 | Spike | A729 | Ser | FCS | 18 |  |
| 24791 | 41 | Spike | T1043 | Ile |  | 19 | X |
| 24820 | 9 | Spike | L1053 | S |  | 19 |  |
| 25197 | 87 | Spike | S1178 | S |  | 20 | X |
| 25521 | 41 | Spike | Y1286 | S | TM | Between |  |
| 25602 | 36 | Spike | T1313 | S | Tail | Between |  |
| 25684 | 87 | ORF3 | S9 | S |  | Between |  |
| 25715 | 41 | ORF3 | R21 | Ser |  | 21 | X |
| 25716 | 41 | ORF3 | R21 | Ser |  | 21 |  |
| 26093 | 87 | ORF4a | D27 | Ala,His |  | 22 | X |
| 26100 | 36 | ORF4a | T29 | Ile |  | 22 |  |
| 26152 | 87 | ORF4a | T46 | S |  | 22 |  |
| 26174 | 87 | ORF4a | S54 | Ala |  | 22 |  |
| 26194 | 9 | ORF4a | A60 | S |  | 22 |  |
| 26269 | 9 | ORF4a | L85 | S |  | 22 |  |
| 26287 | 9 | ORF4a | L91 | S,Val, Phe ins |  | 22 |  |
| 26295 | 9 | ORF4a | N94 | Ser |  | 22 |  |
| 26299 | 87 | ORF4a | R95 | del |  | 22 |  |
| 26305 | 87 | ORF4a | D97 | S |  | 22 |  |
| 26347 | 87 | ORF4a | T112 | S |  | 22 |  |
| 26718 | 116 | ORF4b | G155 | Ser |  | 23 | X |
| 26783 | 41 | ORF4b | L176 | S |  | 23 |  |
| 26807 | 87 | ORF4b | N184 | S |  | 24 | X |
| 26825 | 9 | ORF4b | L190 | S |  | 24 |  |
| 26849 | 9 | ORF4b | L198 | S |  | 24 |  |
| 26852 | 9 | ORF4b | D199 | S |  | 24 |  |
| 26906 | 9 | ORF4b | T217 | S |  | 24 |  |
| 26950 | 87 | ORF4b | G232 | Asp |  | 24 |  |
| 26981 | 9 | ORF4b | P242 | S |  | 24 | X |
| 27008 | 41 | ORF4b | H251 | S |  | 25 | X |
| 27044 | 41 | ORF4b | C263 | S |  | 25 |  |
| 27090 | 41 | ORF5 | P10 | S |  | 25 |  |
| 27109 | 41 | ORF5 | T17 | S |  | 25 |  |
| 27621 | 9 | ORF5 | L187 | S |  | 25 |  |
| 28122 | 41 | M | T17 | S |  | 25 |  |
| 28125 | 41 | M | E18 | S |  | 25 |  |
| 28134 | 41 | M | F21 | S |  | 25 |  |
| 28149 | 41 | M | L26 | S |  | 25 |  |
| 28152 | 41 | M | F27 | S |  | 25 |  |
| 28223 | 41 | M | S51 | Leu |  | 25 |  |
| 28305 | 116 | M | I78 | S |  | 26 | X |
| 29075 | 41 | N | P96 | S |  | 27 | X |
| 29111 | 41 | N | E108 | S |  | 27 |  |
| 29199 | 41 | N | R138 | S |  | 27 |  |
| 29354 | 41 | N | R189 | S |  | 27 |  |
| 29357 | 41 | N | S190 | S |  | 27 |  |
| 29360 | 41 | N | N191 | S |  | 27 |  |
| 29361 | 41 | N | A192 | Ser |  | 27 |  |
| 29396 | 41 | N | G203 | S |  | 27 |  |
| 29585 | 41 | N | F266 | S |  | 27 |  |
| 29681 | 41 | N | E298 | S |  | 27 |  |
| 29711 | 41 | N | S308 | S |  | 27 |  |
| 29768 | 41 | N | Y327 | S |  | 27 |  |
| 29780 | 41 | N | Y331 | S |  | 27 |  |
| 29987 | 41 | N | V400 | S |  | 27 |  |
| 30113 | 41 | 3'UTR | N/A | N/A |  | 27 |  |
