# Supplementary material for: Recombination alters the receptor binding and furin cleavage site in novel bat-borne HKU5-CoV-2 coronavirus
Source: Microbiol Spectr. 2025 Aug 29;13(10):e01420-25. doi: 10.1128/spectrum.01420-25 (PMC12502745; doi:10.1128/spectrum.01420-25)
Supplement: Table S4 — SNP numbers associated with the numbers above the haploblocks for Figure S1. [file spectrum.01420-25-s0003.rtf]

Number above the haploblock in Figure S1	SNP number	
1	127	
2	191	
3	246	
4	247	
5	266	
6	363	
7	385	
8	447	
9	449	
10	471	
11	495	
12	504	
13	522	
14	597	
15	612	
16	630	
17	645	
18	663	
19	750	
20	759	
21	771	
22	777	
23	852	
24	867	
25	903	
26	1090	
27	1113	
28	1121	
29	1155	
30	1167	
31	1184	
32	1218	
33	1230	
34	1353	
35	1393	
36	1396	
37	1404	
38	1476	
39	1491	
40	1492	
41	1524	
42	1542	
43	1560	
44	1581	
45	1619	
46	1620	
47	1638	
48	1683	
49	1773	
50	1779	
51	1818	
52	1821	
53	1863	
54	1908	
55	1930	
56	2001	
57	2040	
58	2052	
59	2062	
60	2076	
61	2077	
62	2110	
63	2148	
64	2163	
65	2177	
66	2182	
67	2183	
68	2190	
69	2208	
70	2221	
71	2223	
72	2233	
73	2244	
74	2256	
75	2259	
76	2307	
77	2464	
78	2487	
79	2490	
80	2493	
81	2547	
82	2569	
83	2688	
84	2716	
85	2819	
86	2949	
87	3027	
88	3034	
89	3117	
90	3150	
91	3275	
92	3312	
93	3438	
94	3470	
95	3488	
96	3503	
97	3567	
98	3651	
99	3671	
100	3683	
101	3743	
102	3746	
103	3828	
104	3831	
105	4002	
106	4215	
107	4257	
108	4509	
109	4573	
110	4660	
111	4672	
112	5025	
113	5052	
114	5091	
115	5142	
116	5400	
117	5552	
118	5581	
119	5658	
120	5761	
121	5770	
122	5911	
123	5922	
124	5931	
125	5969	
126	5970	
127	6028	
128	6039	
129	6072	
130	6141	
131	6146	
132	6159	
133	6177	
134	6184	
135	6201	
136	6204	
137	6213	
138	6365	
139	6379	
140	6389	
141	6397	
142	6400	
143	6401	
144	6414	
145	6492	
146	6543	
147	6690	
148	6692	
149	6815	
150	6872	
151	6882	
152	6933	
153	6951	
154	7091	
155	7092	
156	7119	
157	7138	
158	7158	
159	7182	
160	7200	
161	7366	
162	7491	
163	7494	
164	7539	
165	7692	
166	7826	
167	7869	
168	7870	
169	7986	
170	8289	
171	8400	
172	8418	
173	8463	
174	8482	
175	8508	
176	8523	
177	8544	
178	8571	
179	8589	
180	8631	
181	8676	
182	8685	
183	8719	
184	8748	
185	8799	
186	8805	
187	8817	
188	8835	
189	8848	
190	8892	
191	8997	
192	9128	
193	9201	
194	9234	
195	9252	
196	9501	
197	9621	
198	9667	
199	9706	
200	9790	
201	9869	
202	9892	
203	9903	
204	10074	
205	10233	
206	10317	
207	10344	
208	10366	
209	10392	
210	10446	
211	10668	
212	10689	
213	10731	
214	11163	
215	11184	
216	11259	
217	11433	
218	11565	
219	11619	
220	11721	
221	11790	
222	11985	
223	12246	
224	12330	
225	12342	
226	12480	
227	12588	
228	12678	
229	13032	
230	13159	
231	13298	
232	13446	
233	13560	
234	13578	
235	13753	
236	13859	
237	13874	
238	13997	
239	14051	
240	14059	
241	14111	
242	14185	
243	14279	
244	14288	
245	14294	
246	14360	
247	14364	
248	14372	
249	14453	
250	14454	
251	14585	
252	14783	
253	14849	
254	14870	
255	14873	
256	14918	
257	14972	
258	15044	
259	15101	
260	15353	
261	15485	
262	15503	
263	15566	
264	15743	
265	15755	
266	15764	
267	15836	
268	15965	
269	15977	
270	15998	
271	16004	
272	16088	
273	16112	
274	16125	
275	16223	
276	16235	
277	16238	
278	16274	
279	16289	
280	16313	
281	16322	
282	16349	
283	16377	
284	16427	
285	16433	
286	16460	
287	16481	
288	16604	
289	16637	
290	16676	
291	16734	
292	16772	
293	16797	
294	16841	
295	16874	
296	16994	
297	17018	
298	17054	
299	17057	
300	17072	
301	17102	
302	17162	
303	17174	
304	17189	
305	17198	
306	17204	
307	17213	
308	17507	
309	17721	
310	17921	
311	17993	
312	18065	
313	18118	
314	18287	
315	18397	
316	18499	
317	18728	
318	18791	
319	18824	
320	18887	
321	18911	
322	19013	
323	19019	
324	19115	
325	19205	
326	19292	
327	19322	
328	19418	
329	19499	
330	19572	
331	19896	
332	19973	
333	20078	
334	20358	
335	20396	
336	20513	
337	20555	
338	20603	
339	20633	
340	20645	
341	20933	
342	21053	
343	21081	
344	21209	
345	21215	
346	21581	
347	21670	
348	21671	
349	21726	
350	21729	
351	21735	
352	21737	
353	21738	
354	21740	
355	21741	
356	21742	
357	21753	
358	21766	
359	21767	
360	21774	
361	21777	
362	21780	
363	21781	
364	21782	
365	21786	
366	21788	
367	21789	
368	21798	
369	21800	
370	21801	
371	21804	
372	21810	
373	21812	
374	21829	
375	21831	
376	21837	
377	21840	
378	21841	
379	21849	
380	21852	
381	21879	
382	21882	
383	21897	
384	21939	
385	22017	
386	22021	
387	22023	
388	22038	
389	22047	
390	22062	
391	22065	
392	22068	
393	22072	
394	22073	
395	22110	
396	22122	
397	22122	
398	22162	
399	22164	
400	22167	
401	22170	
402	22176	
403	22182	
404	22185	
405	22188	
406	22191	
407	22200	
408	22206	
409	22215	
410	22224	
411	22232	
412	22234	
413	22236	
414	22237	
415	22238	
416	22239	
417	22245	
418	22264	
419	22272	
420	22273	
421	22275	
422	22281	
423	22290	
424	22296	
425	22297	
426	22299	
427	22311	
428	22314	
429	22315	
430	22316	
431	22320	
432	22323	
433	22326	
434	22329	
435	22332	
436	22344	
437	22356	
438	22369	
439	22371	
440	22378	
441	22395	
442	22398	
443	22404	
444	22412	
445	22434	
446	22437	
447	22449	
448	22452	
449	22458	
450	22473	
451	22479	
452	22488	
453	22494	
454	22495	
455	22498	
456	22533	
457	22539	
458	22542	
459	22545	
460	22548	
461	22551	
462	22554	
463	22555	
464	22556	
465	22557	
466	22559	
467	22560	
468	22578	
469	22597	
470	22614	
471	22617	
472	22618	
473	22620	
474	22662	
475	22674	
476	22698	
477	22704	
478	22713	
479	22714	
480	22722	
481	22734	
482	22749	
483	22765	
484	22782	
485	22783	
486	22803	
487	22824	
488	22827	
489	22848	
490	22851	
491	22854	
492	22863	
493	22905	
494	22911	
495	22914	
496	22933	
497	22938	
498	22956	
499	22959	
500	23001	
501	23016	
502	23031	
503	23033	
504	23034	
505	23043	
506	23064	
507	23100	
508	23104	
509	23122	
510	23130	
511	23151	
512	23155	
513	23156	
514	23160	
515	23171	
516	23172	
517	23175	
518	23178	
519	23193	
520	23196	
521	23199	
522	23205	
523	23212	
524	23214	
525	23223	
526	23226	
527	23238	
528	23241	
529	23250	
530	23263	
531	23265	
532	23267	
533	23272	
534	23285	
535	23340	
536	23343	
537	23349	
538	23352	
539	23355	
540	23358	
541	23368	
542	23371	
543	23373	
544	23377	
545	23379	
546	23383	
547	23384	
548	23385	
549	23387	
550	23389	
551	23394	
552	23395	
553	23403	
554	23406	
555	23412	
556	23413	
557	23418	
558	23421	
559	23425	
560	23427	
561	23428	
562	23430	
563	23433	
564	23438	
565	23439	
566	23442	
567	23443	
568	23445	
569	23451	
570	23460	
571	23463	
572	23465	
573	23466	
574	23467	
575	23476	
576	23478	
577	23479	
578	23480	
579	23487	
580	23517	
581	23523	
582	23526	
583	23538	
584	23541	
585	23547	
586	23562	
587	23568	
588	23586	
589	23587	
590	23589	
591	23616	
592	23619	
593	23625	
594	23631	
595	23634	
596	23637	
597	23643	
598	23651	
599	23676	
600	23682	
601	23699	
602	23700	
603	23704	
604	23707	
605	23708	
606	23710	
607	23718	
608	23724	
609	23733	
610	23749	
611	23757	
612	23763	
613	23766	
614	23778	
615	23779	
616	23780	
617	23781	
618	23784	
619	23793	
620	23799	
621	23817	
622	23823	
623	23826	
624	23830	
625	23833	
626	23841	
627	23843	
628	23847	
629	23847	
630	23848	
631	23860	
632	23862	
633	23877	
634	23878	
635	24006	
636	24159	
637	24186	
638	24195	
639	24225	
640	24234	
641	24252	
642	24276	
643	24294	
644	24414	
645	24474	
646	24585	
647	24621	
648	24663	
649	24738	
650	24747	
651	24791	
652	24813	
653	24820	
654	24861	
655	24909	
656	24957	
657	25116	
658	25149	
659	25197	
660	25203	
661	25215	
662	25230	
663	25242	
664	25274	
665	25308	
666	25395	
667	25419	
668	25521	
669	25602	
670	25684	
671	25715	
672	25716	
673	25733	
674	25789	
675	26093	
676	26100	
677	26117	
678	26128	
679	26152	
680	26164	
681	26166	
682	26174	
683	26179	
684	26194	
685	26240	
686	26269	
687	26280	
688	26281	
689	26282	
690	26287	
691	26295	
692	26299	
693	26305	
694	26347	
695	26489	
696	26523	
697	26525	
698	26714	
699	26718	
700	26724	
701	26747	
702	26762	
703	26765	
704	26783	
705	26794	
706	26802	
707	26804	
708	26807	
709	26825	
710	26849	
711	26852	
712	26885	
713	26906	
714	26950	
715	26973	
716	26981	
717	27008	
718	27038	
719	27038	
720	27044	
721	27090	
722	27108	
723	27109	
724	27252	
725	27402	
726	27444	
727	27453	
728	27497	
729	27517	
730	27573	
731	27621	
732	27623	
733	27678	
734	27680	
735	28086	
736	28122	
737	28125	
738	28134	
739	28149	
740	28152	
741	28223	
742	28251	
743	28305	
744	28428	
745	28828	
746	28868	
747	28988	
748	29075	
749	29111	
750	29199	
751	29336	
752	29354	
753	29357	
754	29360	
755	29361	
756	29396	
757	29414	
758	29585	
759	29648	
760	29681	
761	29711	
762	29768	
763	29780	
764	29987	
765	30113	
